# F108. PSYCHOTIC EXPERIENCES IN A NORWEGIAN SAMPLE - TENTATIVE RESULTS OF A QUESTIONNAIRE VALIDATION

**DOI:** 10.1093/schbul/sby017.639

**Published:** 2018-04-01

**Authors:** Isabella Kusztrits, Frank Larøi, Julien Laloyaux, Lynn Marquardt, Igne Sinkeviciute, Eirik Kjelby, Erik Johnsen, Josef Bless, Kristiina Kompus, Kenneth Hugdahl, Marco Hirnstein

**Affiliations:** 1 University of Bergen; 2 University of Bergen, NORMENT Center for the Study of Mental Disorders, University of Liège; 3 Haukeland University Hospital, NORMENT Center for the Study of Mental Disorders; 4 University Hospital; 5 Haukeland University Hospital, University of Bergen, University of Oslo; 6 University of Bergen, NORMENT Center for the Study of Mental Disorders

## Abstract

**Background:**

Auditory verbal hallucinations are a major symptom in schizophrenia but also affect patients with other diagnoses and healthy people without any pathology. This applies also to delusions and hallucinations of other sensory modalities. Since most questionnaires that assess hallucinations focus on one particular disorder, the Questionnaire for Psychotic Experiences (QPE) was created to provide an instrument that is applicable independently of clinical status.

The QPE is a semi-structured interview consisting of 50 items that are categorized into four subscales (visual, auditory, other hallucinations and delusions). Each subscale starts with a screening question to indicate if a symptom is present, followed by questions regarding specific characteristics.

We translated the QPE into Norwegian (bokmål) and distributed the screening questions online with the aims (1) to validate the Norwegian version of the screener QPE and (2) to assess the prevalence of hallucinations and delusions in the Norwegian population independently of the clinical status.

**Methods:**

We conducted an online survey using a test/re-test design, which comprised the 13 screening questions of the QPE as well as demographic and clinical questions. Seven days after initial completion of the QPE participants received a link for the second round. For test/re-test reliability, we calculated concordance rates (i.e., percentage rates of how many participants gave the same response at the first and second measurement). Internal consistency is indicated with Cronbach’s alpha. Finally, we calculated a principal component analysis (PCA) for the QPE items to identify the QPE’s item structure. The study was approved by the regional ethics committee.

**Results:**

Until now, 407 individuals (304 females, 103 males) with an age range of 18 to 78 (mean = 32.7) participated in the first part of the online survey, of whom 185 also took part in the re-test.

Twenty-eight % of all participants had at least one psychiatric diagnosis. Among the healthy participants alone, 35% reported auditory hallucinations, 26% visual hallucinations, 40% tactile hallucinations and 28% olfactory hallucinations. Around 68% of all healthy participants reported at least one delusional experience.

Cronbach’s alpha across all 13 items for the entire sample was 0.772 in the first round and 0.765 in the second round. Test/re-test reliability was between 79% and 99%. The PCA, also based on the entire sample, revealed one dimension, with high loadings especially on delusion-related questions (range: 0.488–0.697).

**Discussion:**

The distribution of different modalities of hallucinations and delusions in the healthy sample suggests that psychotic experiences are not necessarily connected to diagnoses. This finding is in accordance with other studies and supports the hypothesis that psychotic experiences are independent of the clinical status.

The Cronbach’s alpha suggests a good internal consistency at both time points, which stays stable over time and the test/re-test reliability shows a high accordance between the answers of round one and two. The PCA implies that the QPE screener is best characterized with a unidimensional structure, indicating that there is substantial overlap between hallucinations and delusions, even though factor loadings are particularly high for delusions. We conclude that the Norwegian version of the screener QPE is a viable tool for assessing psychotic experiences across both psychiatric and healthy populations.

